# New Moms Wellness Study: the randomized controlled trial study protocol for an intervention study to increase fruit and vegetable intake and lower breast cancer risk through weekly counseling and supplemental fruit and vegetable box delivery in breastfeeding women

**DOI:** 10.1186/s12905-022-01967-9

**Published:** 2022-09-24

**Authors:** Susan R. Sturgeon, Lindiwe Sibeko, Raji Balasubramanian, Kathleen F. Arcaro

**Affiliations:** 1 Department of Biostatistics and Epidemiology, School of Public Health and Health Sciences, University of Massachusetts, Amherst, MA 01003 USA; 2 Department of Nutrition, School of Public Health and Health Sciences, University of Massachusetts, Amherst, MA 01003 USA; 3 Department of Veterinary and Animal Sciences, College of Natural Sciences, University of Massachusetts, Amherst, MA 01003 USA

**Keywords:** Fruit, Vegetables, Diet, Inflammatory markers, DNA methylation, Breast cancer

## Abstract

**Background:**

Laboratory studies indicate that chemicals in fruits and vegetables have anti-carcinogenic and anti-inflammatory activities that can lower breast cancer risk. However, epidemiologic studies of the association between fruit and vegetable intake and breast cancer risk have produced mixed results. Measurement error, confounding, and an emphasis on diet in later adulthood may contribute to weak associations. This paper describes a randomized controlled diet intervention trial in breastfeeding women to examine the effect of high fruit and vegetable intake on breast cancer risk factors, including weight, DNA methylation and inflammatory markers.

**Methods:**

Eligible breastfeeding women who reside within a 35-mile radius of Amherst, MA are enrolled at five to six weeks postpartum and randomly assigned to a Fruit and Vegetable Intervention Arm (target n = 200) or to a USDA MyPlate Control Arm (target n = 200). The Fruit and Vegetable Intervention group receives weekly telephone or video-based counseling to encourage intake of at least eight to ten daily servings of fruits and vegetables and a weekly delivery of a supplemental box of fruits and vegetables for 20 weeks, and less intensive counseling for up to one year. Breastmilk and infant fecal specimens are collected at baseline, 10 and 20 weeks. Anthropometric measurements are obtained at these timepoints and at the 1-year follow-up. The primary outcomes are change in DNA methylation in breast epithelial cells and change in inflammatory markers in breastmilk from randomization to 20 weeks; and change in weight, waist circumference, and fruit and vegetable intake for the period from randomization to 20 weeks and 1 year.

**Discussion:**

This 1-year randomized diet intervention trial in breastfeeding women will assess whether intake of at least eight to ten daily servings of fruits and vegetables per day improves biomarkers of breast cancer risk directly in the breast (i.e., DNA methylation and inflammatory markers) and helps women maintain a healthy weight.

*Trial registration*: ClinicalTrials.gov Identifier: NCT04374747. Registered May 5, 2020. https://www.clinicaltrials.gov/ct2/show/NCT04374747.

## Background

Compounds in fruits and vegetables have anti-carcinogenic and anti-inflammatory activity that may lower breast cancer risk [1]. However, studies of the association between fruit and vegetable intake and breast cancer risk have produced mixed results. The 2017 American Institute for Cancer Research report concluded that there is some evidence that non-starchy vegetables and carotenoid-rich foods reduce risk [1]. Higher vegetable intake may be more strongly associated with a reduction in estrogen receptor (ER)-negative breast cancer [2]. Most prior studies have been observational in design with a focus on diet in later adulthood. Therefore, we will address a significant gap in the literature by conducting a randomized trial in young women to assess the effect of consuming up to 8 to 10 daily servings of fruits and vegetables on biomarkers of breast cancer risk in the breast microenvironment and helps women maintain a healthy weight. We hypothesize that high fruit and vegetable intake in this population will favorably impact DNA methylation patterns and the inflammatory profile in the breast.

Global DNA hypomethylation and hypermethylation of tumor suppressor genes are key early events in breast carcinogenesis [3]. Aberrant DNA methylation patterns in breast cells are highly promising molecular biomarkers of breast cancer risk [4]. Chronic inflammation may also be involved in breast cancer development and progression. Many prospective studies have examined the relation between higher blood levels of c-reactive protein (CRP), and risk of breast cancer, and the overall findings indicate a modest positive association [5]. While the weight of evidence supports a role of inflammation in the development of breast cancer, the weak associations in epidemiologic studies have been attributed to the focus on circulating inflammatory markers. There is considerable evidence indicating that serum is not adequate surrogate tissue for understanding local inflammatory activity in the breast and breast cancer risk [6].

Our intervention study population is breastfeeding women because the risk of breast cancer is increased for many years after childbirth [7]. In addition, a dietary intervention to reduce breast cancer may be more effective in younger than in older women. Finally, breast milk provides access to the breast to study DNA methylation and inflammation.

Although DNA methylation is considered stable, environmental exposures including diet, can alter methylation patterns [8–10]. Most studies of diet and epigenetics have focused on foods that supply nutrients (I.e., folic acid and B vitamins) for the one-carbon metabolic pathway because of the pathway’s role in supplying methyl groups needed in the DNA methylation reaction [10]. Direct dietary supplementation with foods aimed at enriching the methyl pool is just one way diet can alter DNA methylation. Compounds in fruits and vegetables (e.g., polyphenols, Vitamin C, lycopene, isothiocyanates) may also decrease methylation by inhibiting DNA methyltransferases, or by acting as cofactor to demethylation enzymes [9, 10]. Several prior studies have shown an intriguing association between diets rich in fruits and vegetables and DNA methylation patterns. For example, a prudent diet high in fruits and vegetables was associated with a lower prevalence of global hypomethylation measured in white blood cell (WBC) DNA than a western diet [11]. In another study, a four-week intervention in which participants were randomized to one of three diet groups (balanced fruits and vegetables, a diet rich in flavonoids and isothiocyanates, or a diet supplemented with flavonoids) showed that all three interventions resulted in a small but reproducible decrease in hypomethylation of WBC DNA as compared to pre-intervention levels [12]. Another study reported that diets containing 300 g of vegetables and legumes plus hazelnut oil for eight weeks resulted in a favorable reduction in methylation of *ADRB3* in blood of overweight and obese women without weight loss [13]. Intriguingly, DNA methylation was also altered in nipple aspirate cells of women participating in an intervention study with isoflavones after one month [14]. In summary, DNA methylation is a key event in carcinogenesis and diet, particularly fruit and vegetable intake, may be an effective way to shift DNA methylation patterns to lower breast cancer risk, yet the effect of a diet rich in fruits and vegetables has not been assessed directly in breast tissue.

The role of intake of fruits and vegetables in reducing inflammation in the breast is unknown. Several dietary intervention studies (ranging from 3 months to 2 years in length) have suggested that a dietary pattern high in fruits and vegetables is associated with increased blood levels of adiponectin, and lower blood levels of IL-6, and CRP [15]. For example, Watzl and colleagues reported a significant decrease in CRP levels after participants consumed 8 daily servings of vegetables for 4 weeks [16]. However, understanding the potential effects of a diet rich in fruits and vegetables on breast tissue levels of inflammatory markers is more relevant to breast cancer susceptibility.

Therefore, we are conducting a 1-year randomized diet intervention trial (parallel design, 1:1 allocation, superiority framework) in breastfeeding women to assess the extent to which intake of at least eight to ten daily servings of fruits and vegetables improves biomarkers of breast cancer risk (i.e., DNA methylation and inflammatory markers) and helps women maintain a healthy weight.

### Aims

Specific aims for mothers are: (1) to determine the extent to which a diet intervention high in fruits and vegetables, compared to a control diet alters breast epithelial cell DNA methylation, breastmilk inflammatory profiles and maternal weight and waist circumference at 10 and 20 weeks; and (2) at 20 weeks and the 1-year interval, determine the extent to which randomization to the 1-year diet intervention, compared to the control diet, helps mothers increase fruit and vegetable consumption and maintain a healthy weight and body fat distribution.

## Methods

### Overview

Participants for the New Moms Wellness Study will be recruited from both pregnant women who plan to breastfeed and recently postpartum breastfeeding women who reside within a 35-mile radius of Amherst, Massachusetts. Eligible women will be enrolled at five to six weeks postpartum and randomly assigned to a Fruit and Vegetable Intervention Arm (target n = 200) or to Control Arm (target n = 200). The Fruit and Vegetable Intervention Arm will receive weekly telephone or video counseling and a weekly home delivery of supplemental fruits and vegetables for 20 weeks, and less intensive counseling for up to one year. All women will have access to a lactation counselor during the study. Remote study visits will occur at baseline, 10 weeks, 20 weeks and one year. Annual follow-up will continue for up to three years.

Data will be collected using REDCap hosted at University of Massachusetts Worcester [17, 18].

Although our original study design involved four home visits with in-person measurements (anthropometrics and skin carotenoid levels), the onset of the SARS-CoV-2 pandemic necessitated adopting a fully remote design as described below.

### Eligibility criteria

Eligible women will be women five to six weeks postpartum who are breastfeeding and over 18 years of age, who reside within 35 miles of Amherst MA or Worcester MA, and who have a National Cancer Institute Fruit and Vegetable Screener score indicating five or fewer servings of fruit and vegetables consumed per day over the past month [19]. Women with the following characteristics will be excluded: prior cancer in the past five years; a condition which may interfere with digestion and absorption of nutrients, body mass index < 18.5 kg/m^2^ or a personal history of diabetes (excluding gestational diabetes). An eligibility checklist will be administered at the time of first contact (up to 9 months prior to enrollment) and this requirement will be included as part of the informed consent process. The fruit and vegetable screener will not re-administered as we consider fruit and vegetable consumption to be relatively stable and most newly postpartum women do not consume more than five servings of fruits and vegetables per day.

### Recruitment

Potential study participants will primarily contact us though our website portal, having learned of the study through social media (including new mother groups, community listservs, and Facebook sponsored advertisements), print and electronic brochures at pediatric, midwifery, obstetric offices, birth classes, breastfeeding classes, Women Infant and Children’s (WIC) offices, community baby showers, and local businesses. Some participants will be recruited by a few practices or businesses that briefly describe the study to clients and will collect contact information from interested study participants that will then be shared with us for follow-up. All recruitment and study materials will be translated into Spanish.

### Randomization

All participants will provide written informed consent. Randomization will occur shortly after the baseline visit by study personnel. We will use block randomization (1:1 allocation) by self-reported body mass index (kg/m^2^) [18.5–24.9, 30+) and self-reported race/ethnicity (White non-Hispanic, White Hispanic, Black, Non-Hispanic or Black Hispanic, Other). The allocation sequence will be created by the biostatistician using computer-generated random numbers, and implementation will be through REDCap.

### Fruit and vegetable intervention arm

During the first 20 weeks of the intervention, participants will receive weekly produce boxes providing 32 servings of fruits and vegetables. The boxes will be comprised of 75% vegetables, with at least six servings of leafy greens. To encourage intake of variety of fruits and vegetables, a packing guide for produce boxes will be created based on nutrients and phytochemicals groupings developed by and Fisher (Table 1) [20]. At least one item from each category will be packed into the supplemental box of produce, which will be delivered to each participant along with recipes to assist in meal preparation. These boxes of fruits and vegetables will assist participants to transition from their pre-study diet and are intended to help the intervention participants achieve their goal of consuming 8 to 10 servings of fruits and vegetables, but not to supply their total fruit and vegetable intake or to restrict other produce intake. The produce box will contain a combination of fresh and frozen fruits and vegetables, providing participants with food preparation options. Participants will not be deterred from consuming canned produce, since research indicates canned and frozen fruits and vegetables are nutritionally sound alternatives to fresh produce [21–23]. The content of weekly boxes will be modified based on availability and study participant allergies.

**Table 1:** Study activities at each assessment

	Pre-enrollment	Baseline	10 weeks	20 weeks	1 year
*Eligibility*					
Informed consent	X				
Eligibility Screener	X				
Fruit and vegetable screener	X				
*Randomization*					
Randomization		X			
Nutrition education session		X			
*Intervention arm only*					
Fruit and vegetable delivery (weekly for 20 weeks)		X	X	X	
Dietary counseling (weekly for 20 weeks then monthly up to 1 year)		X	X	X	
Three 24-Hour Diet Records (weekly for 20 weeks to guide counseling)		X	X	X	
Monthly counseling check-ins (between 20 weeks and one year)				X	X
*Anthropometrics*					
Maternal height	X	X			
Maternal weight	X	X	X	X	X
Maternal waist circumference		X	X	X	X
Infant length and weight		X	X	X	X
*Biological sample collection*					
Breastmilk specimen (if still breastfeeding)		X	X	X	X
Infant fecal specimen		X	X	X	X
Maternal fecal specimen (optional)		X		X	X
*Questionnaire data*					
Three 24 hour diet recalls (ASA24)		X	X	X	X
Breastfeeding Practices Questionnaire		X			
Breastfeeding Update Questionnaire			X	X	X
New Moms Health Questionnaire		X			
Medication and Supplement Questionnaire		X	X	X	X
Infant and Child Feeding Questionnaire		X	X	X	X
Physical Activity Questionnaire (IPAQ)		X	X	X	X
Breast Health Questionnaire					X

The dietary intervention group will also receive weekly tailored counseling over the phone to promote intake of 8–10 daily servings of fruits and vegetables. Nutrition counseling and support will occur on a weekly basis from six weeks postpartum until 26 weeks postpartum, when the supplemental produce boxes will be discontinued. At this point, nutrition counseling will continue, with less frequency, up to the end of the 12-month intervention. Individualized counselling will involve supportive and motivational interviewing and goal setting techniques to help participants achieve intervention goals (e.g., modifying recipes and food preparation) [24, 25]. Servings are defined as 1 cup of cut, raw or cooked vegetables or fruit, or two cups of raw leafy vegetables. Fruit juices are not counted toward daily goals, because of their association with weight gain [26] and due to the removal of key nutrients such as fiber.

Participants will monitor their compliance to the intervention by keeping three food records per week (two weekdays and one weekend) and recording, in as much detail as possible, all food, beverages, and supplements consumed in 24 h. These daily records will be collected weekly (for the first 20 weeks), reviewed by the nutrition counselors and used to monitor participant progress. Recording their own intake will also help participants focus on their food choices so they can find ways to increase their fruits and vegetables without disrupting their daily routine. After 20 weeks, the weekly counseling will be guided by a series of questions related to maintaining the study goal of consuming 8 to 10 daily servings of fruits and vegetables per day.

### Control arm

At the baseline visit, nutrition counselors will provide participants randomized to the control group with an overview of the six principles of healthy eating based on the USDA MyPlate Plan [27]. Control participants will also receive a set of MyPlate healthy eating tip sheets by email shortly after the baseline visit which review these principles. We estimate that the USDA Daily Food Plan has the equivalent of four servings per day of the fruits and vegetables specified in our study.

### Study visits

Details on data collected each study visit are described in Table 2. Briefly, there will be four remote study visits: baseline (which occurs at about 6 weeks postpartum), 10 weeks, 20 weeks and at 52 weeks.

**Table 2:** Primary outcome assessments

	Baseline	10 weeks	20 weeks	1 year
12 inflammatory markers	X	X	X	
DNA methylation	X	X	X	X
Maternal weight	X	X	X	X
Maternal weight circumference	X	X	X	X
Fruit and vegetable intake	X	X	X	X

### Primary outcome variables

Table 3 shows the primary outcome assessments, which are described in more detail below.

**Table 3:** Grouping of fruits and vegetables for produce packing

Dark leafy greens	Collards, Kale, Mustard Greens, Romaine, Spinach, Swiss Chard
Lettuce	Butterhead, endive, leaf (red or green) lettuce
Deep orange/yellow fruit, roots or tubers	Apricot, cantaloupe, mango, nectarine, peach, papaya, butternut squash, carrot, Hubbard squash, pumpkin, sweet potato
Red fruit/vegetables	Cherries, pomegranate, watermelon, beets, red pepper, tomato
Citrus	Clementine, grapefruit (red/white), orange, tangerine
Cabbage family	Broccoli, Brussel sprouts, cabbage (green/red), cauliflower, Chinese broccoli, Chinese cabbage, bok choy
Allium	Garlic, leek, onion, scallion, shallot
Red/purple/blue berries	Cranberries, blackberries, blueberries, raspberries, strawberries
White (flesh)	Apple, pear, banana, cucumber, mushroom, crookneck (summer) squash, zucchini, parsnips, radish, turnip, jicama, rutabaga, potato
Unique fruit/vegetables	artichoke, fig, grapes, honeydew, kiwi, pineapple, plum, asparagus, avocado, celery, corn, eggplant, green pepper, okra, snap beans (green beans), snow peas

#### Inflammatory markers

We will use Mesoscale Discovery electrochemiluminescent sandwich assays to determine concentrations of 12 inflammatory markers in bilateral breastmilk samples collected at baseline and 20 weeks (adiponectin, leptin, c-reactive protein (CRP), interferon-γ (IFN-γ), interleukin-1β (IL-1β), interleukin-6 (IL-6), interleukin-8 (IL-8), tumor necrosis factor-α (TNF-α), basic fibroblast growth factor (bFGF), fms related receptor tyrosine kinase 1 (FLT1), placental growth factor (PlGF), vascular endothelial growth factor-D (VEGF-D) as described in a previous study [28].

#### DNA methylation

We will use the Infinium MethylationEPIC Kit (Illumina) to assess DNA methylation in epithelial cells from bilateral breastmilk samples collected at baseline and 20 weeks as described in a previous study [29].

#### Anthropometrics

Maternal and infant anthropometric measurements will be done at baseline, 10 weeks, 20 weeks and at 52 weeks as follows to accommodate COVID-19 pandemic-related contact restrictions.

#### Maternal weight

Weight in kilograms will be self-reported using a provided scale. Trained study personnel will guide participants in taking their weight measurement. Written instructions will be provided to each participant prior to the appointment. Measurements will be taken in duplicate and averaged.

#### Maternal waist circumference

Waist circumference in centimeters will be self-reported, using a provided tape measure. A video demonstrating appropriate measurement will be provided to the participant before the study visit. A trained study interviewer will guide the participant through the measurement. Measurements will be taken in duplicate and averaged.

#### Assessment of diet, including fruit and vegetable intake

Food intake will be collected using the Automated Self-Administered 24-h (ASA24) Dietary Assessment Tool [30], version ASA24-2020. Estimates of energy, nutrients, fruits and vegetables food group intake and portion size will be assessed using 24-h recall data collected at baseline, 10 weeks, 20 weeks, and 52 weeks. At each time point, two recalls will be obtained on weekdays and one recall on the weekend. For individuals who do not have computer access to high-speed internet, we will administer the ASA24 over the phone.

#### Potential covariates and modifiers

We will collect detailed information at the baseline visit on reproductive history, hormone use, prior pregnancies and breastfeeding practices, tobacco use and vaping, marijuana use, alcohol intake, weight, breast health, multivitamin and supplement use during pregnancy, and family history of breast and ovarian cancer. We will also collect updated information on physical activity, breastfeeding and infant feeding practices, medication and supplement use, and breast health.

### Secondary outcomes

#### Infant weight and length

The weight of the infant-mother dyad will be used to calculate infant weight. First, the mother will be instructed to weigh herself, after which she will be instructed to weigh herself while carrying her infant (lightly clothed with clean diaper). Infant weight will be calculated from the difference between mother plus infant weight minus maternal (only) weight. Measurement will be repeated and averaged.

Butcher paper will be used for measuring infant length. Ideally, the mother will have assistance in positioning the infant on the paper as instructed by the study personnel into a straight recumbent position. A line will be drawn on the paper at the position of the top of the head and at the bottom of the feet position. The length will be measured using the tape measure supplied to each participant. Measurement will be repeated and averaged.

#### Microbiome sample collections for future analyses

We will also collect, process and archive breastmilk, infant fecal specimens and maternal fecal specimens to allow us to examine the microbiome relevant to the health of the infant and mother in future analyses.

### Data analysis

All analyses will follow the intent to treat principle. Analyses will be conducted with group labels that do not map to treatment arm.

#### Changes in inflammatory markers and methylation profiles comparing the intervention arm to control arm

To conserve power for the DNA methylation analysis, we will analyze approximately the most variable 300 K DNA methylation profiles. Each inflammatory and methylation marker will be analyzed in separate linear mixed effects models with a random intercept to account for the within subject correlation of the repeated measurements obtained at baseline and 20 weeks. Prior to analysis, each marker will be natural logarithm transformed to satisfy normality assumptions. Each linear mixed effects model will be of the form: E(Y_ij_) = β_0_ + β_1_ x T_ij_ + β_2_ x G_i_ + β_3_ x G_i_ x T_ij_, where Y_ij_ denotes the CpG or inflammatory marker measurement for subject *i* at visit *j*, T_ij_ is the time (post randomization) for subject *i* at visit *j* and G_i_ denotes the randomization group for subject *i*. The primary hypothesis test of interest is H_0_: β_3_ = 0, testing the interaction of randomization group with time, using a likelihood ratio test. As a secondary analysis, we will also consider a linear model in which the baseline CpG level is included as an additional covariate. Missing data imputation methods will be considered to confirm the robustness of the findings. Adjustment for multiple comparisons will be based on the False Discovery Rate (FDR) procedure of Benjamini and Yekutiel [31], which allows for correlation between CpG or inflammatory marker levels. Threshold for statistical significance will be based on the raw p value that controls the rate of false discoveries to be under 5%. The adjustment for multiple testing will be carried out separately for the set of 300,000 CpG measurements and the set of 12 inflammatory markers.

#### Changes in maternal body weight and body fat distribution comparing the intervention arm to control arm at 20 weeks

Separate multivariate linear regression models will be fit to analyze the effect of randomization group on body weight or body fat distribution (waist circumference) at the end of intervention at 20 weeks. Body weight or waist circumference at 20 weeks will be considered as the outcome, with independent covariates including baseline weight or waist circumference, randomization group and other potential confounders. The distributions of body weight and waist circumference will be examined graphically and appropriate transformations (e.g., natural logarithm) will be considered. The statistical significance of the effect of randomization group on outcome will be assessed through a likelihood ratio test.

#### Changes in FV consumption, maternal body weight and body fat distribution comparing the intervention arm to control arm at 1 year

As described above, separate multivariate linear regression models will be fit to analyze the effect of randomization group on (1) average number of servings of FV consumed, (2) body weight and (3) body fat distribution (waist circumference) at the end of intervention at year 1.

### Power and sample size calculations

Power calculations for linear mixed models with adjustment for multiple testing were carried out through simulation studies. Due to computational considerations, adjustment for multiple testing was carried out through a conservative Bonferroni correction procedure to maintain the overall type I error at 0.05. CpG measurements (1a): In each dataset, we simulated repeated measurements of CpG levels according to a multivariate normal distribution with unit variance, assuming a within-subject correlation ρ, a mean difference in levels in the *control group* comparing baseline to the 20-week (T2) visit equal to 0.1 SD units and a mean difference in the intervention group between the 20-week (T2) and baseline (T0) of Δ SD units. The threshold for statistical significance was set at p = 0.05/300000, corresponding to a Bonferroni correction to maintain the overall Type I error at 0.05. When the correlation between the repeated CpG measurements is set to ρ = 0.5 (ρ = 0.3), a sample size of 200 per group results in 99% (82%) power to detect a mean difference in the intervention arm between baseline and T2 of Δ = 0.75 SD units, obtained by averaging over 100 simulated datasets. For the analysis of the 12 inflammatory markers, we simulated repeated measurements of inflammatory marker levels according to a multivariate normal distribution as described above. Assuming a Bonferroni correction to maintain the overall Type I error at 0.05 and when the correlation between the repeated inflammatory measurements is set to ρ = 0.5 (ρ = 0.3), a sample size of 200 per group results in 94% (84%) power to detect a mean difference in the intervention arm between baseline and T2 of Δ = 0.45 SD units, obtained by averaging over 100 simulated datasets.

Power calculations for the analyses of body weight, waist circumference and fruit and vegetable consumption at 1 year were based on a generalized linear model, using the R package pwr [32]. A sample size of 400 subjects results in 98% power to detect an effect size of 0.05 or larger, assuming a two-sided test and a type I error 0.05. We assumed the same variability at the two time points so the power is the same for 20 weeks and one year.

Previous studies found a reduction of 3.90 kg in the intervention arm compared to the control group in weight change from baseline, with an associated SD of approximately 4.8 kg [33]. A 98% power to detect an effect size of 0.05 or larger would correspond to a difference in weight change of 0.24 kg or larger between the two randomized groups. Previous studies found a reduction of 7.4 cm in the diet-intervention arm compared to the control group in waist circumference change from baseline, with an associated SD of approximately 15 cm [34]. A 98% power to detect an effect size of 0.05 or larger would correspond to a difference in waist circumference change of 0.75 cm between the two randomized groups. Our preliminary data showed a mean increase in fruit/vegetable consumption in the diet intervention arm of 7.3 servings with an associated SD of approximately 3 servings [35]. A 98% power to detect an effect size of 0.05 or larger would correspond to a difference in fruit/vegetable consumption change of 0.15 servings between the two randomized groups.

## Discussion

This innovative trial is designed to test whether weekly dietary counseling to encourage intake of at least eight to ten fruits and vegetables daily and short-term home delivery of a box of fruits and vegetables increases fruit and vegetable intake in postpartum women, and favorably impacts postpartum weight and intermediate biomarkers of breast cancer risk, including DNA methylation and inflammatory markers in the breast microenvironment. Strengths of the study include (1) testing the dietary intervention during a period of breast inflammation and remodeling, (2) use of breastmilk, a vastly underutilized source of biological fluid and epithelial cells to study intermediate biomarkers of breast cancer risk, (3) an intervention focused solely on increasing fruit and vegetable intake to modify weight and other outcomes, (4) weekly counseling by training counselors to increase fruit and vegetable intake, (5) weekly home delivery of supplemental fruits and vegetables, and (6) archived milk and maternal and infant fecal specimens which offer unique future research opportunities (e.g., effect of diet on the mother’s human milk oligosaccharides and effects on the infant’s gut microbiome).

Despite recommendations, few Americans meet the daily recommendations for fruit and vegetable intake [36]. The postpartum time period, with its focus on maternal and infant health, offers an opportune time to intervene and promote a healthy dietary lifestyle. A strong link between fruit and vegetable intake and intermediate biomarkers of breast cancer in lactating women (weight, breast epithelial DNA methylation and inflammation) would strengthen public health recommendations to increase fruit and vegetable consumption to lower breast cancer risk. Furthermore, demonstrating that the combination of dietary counseling and short-term fruit and vegetable home delivery in lactating women results in a substantial and sustained intake of fruits and vegetables could provide a new model for improving maternal diets.

## Data Availability

Not applicable.

## Abbreviations

bFGF: Basic fibroblast growth factor
cm: Centimeter
CRP: C-reactive protein
FLT1: Fms related receptor tyrosine kinase 1
IFN-γ: Interferon-γ
IL-1β: Interleukin 1-β
IL-6: Interleukin 6
IL-8: Interleukin 8
kg: Kilograms
PIGF: Placental growth factor
REDCap: Research Electronic Data Capture
TNF-α: Tumor necrosis factor-α
USDA: United States Department of Agriculture
VEGF-D: Vascular endothelial growth factor D
WBC: White blood cell
